# Instrument for assessing (self)care competence in people with gastrostomy and their caregivers: a methodological study

**DOI:** 10.1590/0034-7167-2024-0484

**Published:** 2025-12-08

**Authors:** Luzia Raquel Lopes Pinheiro, Maria Alice Correia Brito, Célia Samarina Vilaça Brito Santos

**Affiliations:** I Instituto Ciências Biomédicas Abel Salazar. Porto, Portugal; II Escola Superior de Enfermagem do Porto. Porto, Portugal

**Keywords:** Self Care, Enterostomal Therapy, Gastrostomy, Jejunostomy, Nursing, Autocuidado, Estomaterapia, Gastrostomía, Yeyunostomía, Enfermería.

## Abstract

**Objectives:**

to describe the development process of an instrument to monitor the development of (self-)care competencies in individuals with gastrostomies and their caregivers.

**Methods:**

methodological research study. Content validity was obtained through a focus group of experts. The instrument was pre-tested on a sample of 18 participants, using a non-probabilistic and intentional sampling technique. Inter-rater agreement was also assessed at a hospital in northern Portugal.

**Results:**

the study resulted in an instrument with 34 measurement indicators, grouped into six domains, with recognized content validity. In the instrument pre-test, good inter-rater agreement values of 80-100% were obtained.

**Conclusions:**

the instrument proved to be comprehensive and easy to apply in assessing (self)care competency in feeding ostomies, offering potential for future validity investigations.

## RESUMEN

**Objetivos:**

describir el proceso de desarrollo de un instrumento para monitorizar el desarrollo de habilidades de (auto)cuidado en personas con gastrostomía y sus cuidadores.

**Métodos:**

estudio de investigación metodológica. La validez de contenido se obtuvo mediante un grupo focal de expertos. El instrumento se probó previamente en una muestra de 18 participantes mediante un muestreo no probabilístico e intencional. También se evaluó la concordancia entre evaluadores en un hospital del norte de Portugal.

**Resultados:**

el estudio generó un instrumento con 34 indicadores de medición, agrupados en seis dominios, con reconocida validez de contenido. En la prueba previa del instrumento, se obtuvieron buenos valores de concordancia entre evaluadores, del 80-100 %.

**Conclusiones:**

el instrumento demostró ser completo y fácil de aplicar para evaluar la competencia en autocuidado en ostomías de alimentación, lo que ofrece potencial para futuras investigaciones de validez.

## INTRODUCTION

Considered one of the basic human needs, nutrition is essential for the maintenance of life, occurring through the processes of ingestion, digestion, and absorption^(1)^. Among the reasons associated with nutritional imbalances are lesions in the central nervous system, such as stroke, changes in the oral cavity, pharynx and esophagus (malformations, tumors, neoplasms, chemical trauma), paralysis (coma, stroke, cerebral palsy), trauma or inadequate coordination of chewing and swallowing (extensive abdominal surgery, extensive brain injuries), after radical surgery of the face and neck or radiotherapy/chemotherapy in the upper digestive tract^(2)^.

Despite the significant advances made by parenteral nutrition, the enteral route remains the first choice for nutritional support in people with a functioning digestive system but who are unable or unable to eat orally. Clinical studies comparing the effectiveness of parenteral nutrition and enteral nutrition conclude that the enteral route is more effective than the parenteral route^(1)^. Feeding ostomies allow nutritional intake to be maintained in the medium and long term via the enteral route^(2)^.

There are different types of feeding ostomies, with medical diagnosis, clinical situation/general condition and treatment time being some of the aspects that influence the choice of the type of ostomy that best suits a person^(1,2)^. They are mainly indicated in situations where a person is temporarily or permanently prevented from eating orally and is expected to receive enteral nutrition for a prolonged period, normally longer than four weeks^(1-3)^.

Given the increased time required for digestive decompression or nutritional support, a gastrostomy is recommended. In circumstances where a gastrostomy is impossible, a jejunostomy is the preferred alternative^(4)^ (gastrostomies, particularly percutaneous endoscopic ones, are the most common, which is why we will use the term “gastrostomy” in this article). A gastrostomy provides access to the stomach through the abdominal wall. The techniques commonly used to perform a gastrostomy are laparotomy (Witzel, Stamm, and Depage-Janeway gastrostomy) and endoscopy (Push, Pull, and Russell techniques)^(4)^.

Regardless of the type of ostomy, a person is subject to profound changes in their physical image and lifestyle, thus requiring adaptation to their new condition, facing new challenges, with several factors conditioning the adaptation process and facilitating or hindering the transition^(5)^. Self-care competency refers to the complex competencies individuals learn to maintain and promote physical and mental health, significantly influencing individual adaptation and playing a vital role in rehabilitation after ostomy placement. Good levels of self-care are positively correlated with overall health and quality of life^(6)^.

The challenge for nursing is to help a person make a healthy transition to their new health condition, promoting and rebuilding their autonomy in the face of the dependency-generating event, which is the existence of a stoma^(6,7)^. The relevance of nurses’ intervention in caregiver preparation and involvement must also be taken into account, ensuring adequate support for the development of competencies for providing care to a person with a stoma, thus promoting a healthy transition to the caregiver role^(7)^. A significant number of people with gastrostomies are dependent on a caregiver for self-care. People with stomas require a series of specific procedures to guide complex therapeutic regimens, maintain well-being and quality of life over time, control risks, manage symptoms, and reduce the likely incidence of complications^(6)^. Therefore, promoting self-care competency is a relevant issue in nursing care for people with stomas, both in research and clinical practice. This will allow for a better understanding of the factors that affect self-care among people with gastrostomies and the implementation of effective self-care promotion interventions. Methodical monitoring, initiated preoperatively and continued postoperatively and after hospital discharge, promotes self-confidence and capacity in stoma care, enhancing psychosocial adaptation and improving quality of life^(6-8)^. The main international organizations of enterostomal therapist nurses also highlight these aspects, defending the need for more research to support these statements^(9,10)^.

It is essential to create instruments that allow nurses, within the scope of their autonomous interventions, to plan and implement care through structured actions. On the one hand, specific and reliable instruments that assess outcomes are used to contribute to healthy transitions and a better quality of life for people with stoma, but on the other, they add knowledge to the discipline and produce outcome indicators. To date, no instrument has been found to assess (self-)care competency in people with gastrostomies, which is the driving force behind this research study. Therefore, the development of an instrument to assess (self-)care in people with gastrostomies and their caregivers, which can be used by nurses to assess outcomes sensitive to their interventions and monitor behavioral changes in patients throughout the transition process, will be a contribution to the nursing discipline and undoubtedly to quality of care.

## OBJECTIVES

To describe the development process of an instrument to monitor the development of (self-)care competencies in individuals with gastrostomies and their caregivers.

## METHODS

In this subchapter, the methodological stages of this study are described.

### Ethical aspects

The research protocol was submitted for approval by the ethics committee of a central hospital in northern Portugal, with a favorable opinion (document 237/2022). The authorization process followed the ethical principles of research, including the Declaration of Helsinki guidelines. All collected information was treated with strict confidentiality, and access to the data was limited to authorized researchers to ensure its security and integrity. To preserve privacy, no identifiable data, such as names, was recorded, thus ensuring the freedom from harm of participants and ensuring the necessary protection of the collected data.

### Study design, period and location

Given the nature of this research and its objective, a methodological study was developed with a qualitative approach that encompasses the construction of a new measurement instrument essential for knowledge production in the area under study^(11)^. Data were collected between February 1 and March 31, 2023, at a central hospital in northern Portugal. This research is part of a larger study on the development and validity of a form to monitor the “Development of (self)care competencies in people with feeding ostomies and their caregivers”.

### Population, sample, inclusion and exclusion criteria

The study population included individuals with gastrostomies and their caregivers who attend the gastroenterology department of a central hospital in northern Portugal. The inclusion criteria for this population were being 18 years of age or older, agreeing to participate, having a gastrostomy, or being a caregiver for someone with a gastrostomy.

The selected sampling technique was intentional non-probability, which allowed easier access to the population^(11)^.

### Study protocol

The methodological study presented here comprises two major stages: the development and partial validity of the created form. The instrument development and content validity to assess the development of (self)care competencies in people with gastrostomy and their caregivers followed the stages clearly defined by Lobiando-Wood and Haber^(12)^:

#### a) Definition of the concept(s) intended to be measured

Thus, the instrument development began with the definition of the following core concepts: self-care; competency; and gastrostomy. Through an extensive literature review, using the descriptors “Self Care”, “Competency”, and “Gastrostomy” in the MEDLINE, CINHAL, Cochrane Clinical Answers, MedicLatina, Library, Information Science Technology Abstracts, PubMed, and stomal therapy nursing textbooks databases^(13,14)^, each sought to define and operationalize the concept of competence in gastrostomy (self)care. Thus, the concept of competence in gastrostomy (self)care was defined, translating it into the dimensions and respective items that allow it to be measured. A search for measurement instruments in the area under study was conducted, but none were found to date in the field of gastrostomies. The instruments found focus on elimination ostomies.

#### b) Enumeration of instrument items that could be constituted as measurable indicators of the concepts under analysis

For the arrangement and ranking of the result indicators identified in the instrument, two pre-existing instruments were used as a working basis:

- The *Escola Superior de Enfermagem do Porto* (ESEP) *Competência do Autocuidado à Ostomia de Eliminação Intestinal* (CAO-EI) form (assessment of the development of competency in self-care for bowel elimination ostomy)^(15)^;
- The ESEP *Competência do Autocuidado à Ostomia de Ventilação* (CAO-OV) form (assessment of the development of competency in self-care for respiratory ostomy)^(16)^.

With the statistical analysis of results obtained by implementing the *Competência do Autocuidado à Ostomia de Eliminação Intestinal* (CAO-EI) form pilot study in the sample of 180 participants, the form obtained an overall Cronbach’s alpha of 0.93, thus presenting good internal consistency^(15)^.

The CAO-OV reliability was assessed using Cronbach’s alpha coefficient, demonstrating good internal consistency (alpha=0.89) in a sample of 80 participants and recognized content validity^(16)^.

Given the multidimensional nature of competency in gastrostomy (self)care, we created a pilot instrument with 33 indicators, organized into six main dimensions/domains, as in pre-existing instruments^(15,16)^, namely: knowledge; self-monitoring; interpretation; decision-making; execution; and negotiation and use of health resources.

Regarding the measurement scale to assess competency for gastrostomy (self)care, the proposed instrument uses the assignment of an ordinal classification, on a five-point Likert scale, which allows for assessing the results achieved in a quantitative manner and with better specificity.

In the first part of the instrument, an area was added to characterize a person with an ostomy or their caregiver, through sociodemographic variables (such as age, marital status, education and professional situation), clinical variables (clinical diagnosis, type of surgery/procedure) and treatment variables (preoperative nursing consultation, previous contact with other people with gastrostomy).

#### c) Study of the content validity of instrument items

To assess the instrument content validity, we opted for a qualitative methodology, specifically a focus group, which sought to incorporate elements from the COnsolidated criteria for Reporting Qualitative research guide to ensure transparency, methodological rigor, analytical rigor, and contextual clarity. The COnsolidated Criteria for Reporting Qualitative Research checklist consists of 32 items, organized into three domains: research team; study concept; and analysis/results^(17)^.

##### • Research team

The data were collected by a researcher pursuing a doctoral degree in nursing science. The researcher led the discussion group meeting. The researcher received guidance from supervisors, who have experience in qualitative research, on data collection in qualitative studies, and was trained in how to collect data in this study design.

Initial contact was established with participants to introduce the researcher, objectives, and their interest in the study. The researcher had no prior relationship with participants.

Participants were informed that the researcher was conducting the study as part of a larger project to develop and validate an instrument for monitoring (self)care competency.

##### • Study concept

The stated methodological approach was content analysis through focus groups. Focus groups are a methodological strategy in which the researcher interacts and engages with a group of experts in the field under study, seeking their in-depth interpretations and perspectives on the topic under study. It consists of a structured discussion among individuals who are experts in a given subject, centered on group interaction and the researcher’s active role as a discussion facilitator^(11)^. To ensure that participating nurses had experience in the field under study, the following inclusion criteria were defined: currently working in a stomal therapy nursing consultation or gastroenterology service with patients with gastrostomies and their caregivers; having at least five years of experience in these services; and being a professor/researcher in the field of self-care and oncology. They also had to freely and willingly agree to participate in the study. Participants for the focus group were selected until an acceptable number of participants was reached. The sample was non-probabilistic and intentional, resulting from acceptance to participate in the focus group. Ten nurses were invited, and all agreed to participate. No one other than participants and the researcher was present. Thus, the expert group consisted of ten professionals, all female, with an average age of 45, mostly specialized in medical-surgical medicine, and five with postgraduate training in stomal therapy, all with over ten years of experience in the field. The group included two nursing professors (one with research in self-care and the other in oncology and stomal therapy) and seven clinical nurses (four stomal therapy nurses working in the stomal therapy nursing consultation and three nurses from gastroenterology services). They jointly assessed the coherence of each indicator and its representativeness for the concept being assessed. This group of experts was chosen because they were recognized as possessing the highest level of disciplinary knowledge in the field under study and worked in hospitals in northern Portugal. They were invited by telephone, informed of the study’s objective, and all agreed to participate (no formal authorization document was required, as the meeting was held off-site, in the professionals’ free time).

##### • Analysis and results

Thus, the initial version of the instrument and its accompanying completion manual (which we explain below) were previously emailed to all members of the expert group for individual review. They were encouraged to analyze each domain, its respective indicators, and criteria defining the (self-)care competency, indicating whether to maintain, modify, or remove it, and justifying their decision. Subsequently, a meeting was scheduled online for October 23, 2023, due to the common difficulty of in-person availability. This meeting involved a carefully planned and designed conversation, seeking to obtain pertinent information about the area of interest among the evaluators^(11)^. The meeting was led by the researcher. The instrument itself was used as a guide, following the outcome indicators and their respective defining criteria. Each indicator and its respective defining criteria were analyzed individually, only moving forward after complete agreement (100%) among the experts. The data verbalized during the meeting by the participants was transcribed into Microsoft Word^®^; no audio or visual recordings were used during the meeting.

At the end of this meeting, which lasted approximately two hours, all participating experts agreed on the 33 indicators for the different competency domains and the respective defining criteria for each indicator, after introducing some changes to the previously used language. The final instrument, including all the changes and suggestions resulting from the focus group, was shared with all participants via email, requesting responses and receiving positive feedback from all ten participants.

#### 4) Creation of the completion manual for instrument users

To ensure the instrument’s correct and rigorous application, a completion manual was created, detailing the criteria defining each of the indicators within the six domains of (self-)care competency, both theoretically and conceptually. This ensures that the scores assigned to the items truly reflect the defining characteristics of the self-care competency indicators, regardless of who administers the instrument.

#### 5) Pre-testing and pilot testing of the instrument’s items/indicators

When a first version of a new instrument is formulated, it is essential to ensure that it is suitable and that it effectively answers the questions posed by the researcher^(11)^. Thus, a pre-test of a new instrument makes it possible to understand how questions and answers are perceived, avoid terminology and enunciation errors, and also reveal vague terms and possible misunderstandings.

The instrument was then subjected to a pre-test with a group of 18 people, nine people with gastrostomy and nine caregivers, separately, accompanied in the outpatient gastroenterology enteric access consultation. Regarding the pre-test sample size, authors agree among themselves^(18)^ that 15 to 30 respondents are sufficient for the pre-test. This research is the result of a larger study developing and validating a form to monitor the “Development of (Self)Care Competencies in People with Feeding Ostomies and Their Caregivers”, with a final sample size of approximately 180 participants. Therefore, we considered 18 people for the pre-test, corresponding to 10% of the sample. The instrument was administered by the principal investigator to each of the pre-test participants, who were asked to respond to the formulated items with elaborated/commented responses, describing the meaning they attributed to the questions individually, as well as to the instrument as a whole, and also including the conditions of its administration.

The principal investigator observed 15 nursing consultations as a complementary method to the pre-test at the proposed institution, assessing whether the indicators for each domain of (self-)care competency, defined for the pilot instrument, fully met the intended objectives. The investigator used the instrument itself as a guide to verify whether they corresponded in practice to the nursing interventions implemented in the (self-)care competency training of the person with gastrostomy or their caregiver in the nursing consultation setting.

## RESULTS

The gastrostomy (self-)care competency was accepted as a multidimensional concept, integrating six domains with corresponding outcome indicators. The indicators were organized and systematized by the six competency domains (knowledge, self-monitoring, interpretation, decision-making, execution, and negotiation and use of health resources). The order of the domains and their respective indicators proved to facilitate the application of the instrument and were relevant for measuring the construct under analysis.

Thus, with the debate and analysis of the instrument in the discussion group, the changes presented in Chart 1 emerged: use of the term “self-care”; use the term “person with stoma”; use the term “procedure” and “surgery”; use the item “waiting for surgery and procedure”.

**Chart 1:** Changes made to the instrument according to the discussion group

Initial version	Final version
“Self-care”	Self-care: use of the term self-care in the title of the instrument, as this can also be applied to the caregiver.
“Patient with an ostomy”	Person with a stoma.
“Did you have contact with people with an ostomy before surgery?”	“Did you have contact with people with a stoma before surgery/procedure?”: considering that not all feeding ostomies are surgical, and are mostly performed using an endoscopic technique.
“Did you participate in a preoperative stomal therapy nursing consultation?”	“Did you participate in a preoperative nursing consultation?”: (considering that people with feeding ostomies are generally monitored by the nursing team of gastroenterology services, with only a small proportion receiving care during stomal therapy nursing consultations).
“How long ago was the surgery/procedure to construct the feeding stoma performed?”	The item “Waiting for surgery/procedure” was included in the first part of the instrument, in point seven “How long ago was the surgery/procedure performed to construct the feeding stoma”, as the instrument can be used pre-operatively.

The instrument and its accompanying manual were then duly revised with the language suggestions suggested by the focus group. After this analysis and harmonization, the final version of the instrument and its accompanying manual was finalized. The six dimensions of gastrostomy (self-)care competency, indicators, and respective domains of gastrostomy (self-)care competency, which were included in the instrument, were maintained and considered relevant and appropriate for measuring the construct under analysis.

Fifteen nursing consultations were observed to complement pre-test results. During the observation of 15 nursing consultations, it was found that nurses consistently addressed the importance of oral hygiene in patients with gastrostomies, a topic not included in the developed instrument. Therefore, it was necessary to include the item “Refers to the importance of oral hygiene”, adding it to the knowledge domain of the feeding ostomy (self-)care competency.

The domains of the instrument’s (self)care competency are: knowledge; self-monitoring; interpretation; decision-making; execution; negotiation and use of health resources (Chart 2).

**Chart 2:** Domains of the instrument’s (self)care competency and respective indicators

Competency domains	Indicators
Knowledge	Explain what a feeding stoma is. Explain the purpose of a feeding stoma. Explain the characteristics of a feeding stoma. Explain signs of complications associated with a feeding stoma. Explain the devices needed for stoma care. Explain when to replace the dressing/fixation mesh. Recognize the importance of oral hygiene. Explain the resources available in the community for people with ostomies. Identify their needs in terms of knowledge about stoma care.
Self-monitoring	Observe the feeding stoma. Identify the characteristics of the feeding stoma. Identify signs of feeding stoma complications. Record significant events.
Interpretation	Ask detailed questions to find an explanation. Mention the possible causes of stoma feeding complications. Recognize that the results of stoma self-care influence your well-being.
Decision-making	Prioritizes decision-making. Recognizes the potential consequences of their decisions. Prevents ostomy complications. Verbalizes what to do to minimize ostomy complications.
Execution	Performs procedures, ensuring comfort. Performs procedures to ensure aesthetically pleasing and functional results. Manages time during procedures to achieve the best results. Organizes the necessary materials for ostomy care. Washes the peristomal skin. Dries the peristomal skin. Places a non-woven fabric compress on the stoma/fixation mesh. Rotates the tube button 360º according to protocol. Administers feedings correctly. Administers medication correctly. Washes the tube after administering feedings/medications and checks that the cap is closed.
Negotiation and use of health resources	Negotiates the various resources available to support the person with an ostomy. Reaches healthcare services for clarification and/or advice. Reaches healthcare services/nursing promptly for ostomy complications. Assesses the care provided by healthcare services.

The completion manual was developed to clarify and standardize the instrument completion, and this manual will contribute to the study of inter-rater reliability. Thus, this manual aimed to ensure that any educated and trained nurse will achieve the same results when assessing the level of (self)care competency.

To measure the indicators mentioned in the different domains, a Likert-type assessment scale was used, ranging from zero to five, where the higher the score, the greater the demonstrated competency in (self)care. The zero demonstrated “not applicable”; one “does not demonstrate”; two, three and four “partially demonstrates” (according to the percentage of criteria defined for the indicator); and five “fully demonstrates” (Chart 3).

**Chart 3:** Likert scale scoring and interpretation

Likert scale Score	Interpretation
0	Not applicable
1	Does not demonstrate
2	Partially demonstrates: compliance with approximately 25% of the criteria identified for the competency domain indicator
3	Partially demonstrates: compliance with approximately 50% of the criteria defined for the indicator
4	Partially demonstrates: compliance with approximately 75% of the criteria established for the competency domain indicator
5	Fully demonstrates

During the pre-test, a spoken reflection was also carried out with the people to whom the instrument was applied, who did not identify any questions that were less clear or difficult to interpret.

The instrument developed was a form, structurally similar to the questionnaire, but completed by the researcher. Thus, the final form maintains the six dimensions of self-care competency, now including a total of 34 items. The assessment of these 34 items requires the researcher to assess the development of (self)care competency by questioning and observing a person with gastrostomy and their caregiver, considering the indicators related to each domain of the competency.

To study inter-rater agreement, also known as inter-rater reliability, a comparison was made between the data obtained by observer A and those obtained by observer B. Regarding the number of participants for the inter-rater agreement study^(11,18)^, approximately 10% of the sample was defined. The two observers administered the form simultaneously to the same subjects, 14 caregivers and seven people with feeding stoma.

Through the chart analysis, it is possible to verify that there is a strong correlation between the results obtained by the different evaluators, with the self-monitoring, interpretation and decision-making domains obtaining an rs value above 0.80, with a significance level of 0.001 (Chart 4). The domains of “knowledge”, “execution” and “negotiation and use of health resources” obtained an rs value equal to 1, which reflects a perfect correlation (Chart 4).

**Chart 4:** Assessment of inter-rater agreement using the non-parametric Spearman’s correlation test

	Knowledge Evaluator B	Self-monitoring Evaluator B	Interpretation Evaluator B	Decision-making Evaluator B	Execution Evaluator B	Negotiation Evaluator B
Knowledge Evaluator A	1.000^**^					
Self-monitoring Evaluator A		0.896^^**^^ ^p<0.001^				
Interpretation Evaluator A			0.802^^**^^ ^p<0.001^			
Decision-making Evaluator A				0.847^**^ ^p<0.001^		
Execution Evaluator A					1.000^**^	
Negotiation Evaluator A						1.000^**^

## DISCUSSION

People with gastrostomies must be prepared for self-care, aiming for their empowerment and autonomy. Empowering people with gastrostomies and their caregivers can directly impact cost reduction and complications^(19)^. Therefore, nurses must involve a person and/or caregiver, whenever possible, in all care provided^(19)^. It is known that practical training in gastrostomy (self)care competencies, support and advice from nurses help to effectively face the challenges faced by people and their caregivers^(20)^.

The scarce scientific literature on the specific assessment of the self-care competency of people with gastrostomies and their caregivers prompted investment in the development of this instrument. Nurses will thus be able to collect accurate and comprehensive data, identifying the difficulties experienced by people with gastrostomies and their caregivers in the area of self-care competency. This instrument has proven capable of assessing needs in the area of self-care competency, allowing nurses to identify real areas of focus and interventions tailored to the continuous improvement of nursing care.

The first part of the instrument, which integrates sociodemographic data and clinical and treatment variables, was considered relevant for subsequent analysis of its relationship with the main variable: (self)care competency. These characteristics should be known, as they may be related to the progress of self-care competency and, in the future, allow the development of research hypotheses.

We assume the concept of (self)care competency as multidimensional, integrating six dimensions (knowledge, self-monitoring, interpretation, decision-making, execution, and negotiation and use of health resources)^(15,16)^. These domains and respective indicators translate the concept we intend to measure.

The instrument can be considered lengthy, including 34 indicators. However, this is reflected in the need for comprehensiveness to encompass a broad set of indicators, sufficient to ensure proper monitoring of the development of this competency.

After the meeting with the group of experts, it was possible to reformulate the instrument, thus ensuring the congruence of each indicator and its representativeness in the concept to be assessed.

Regarding inter-rater reliability, when comparing the data obtained by two different evaluators, perfect or high positive correlations were observed, reflecting high agreement. The instrument’s completion manual was crucial for direct observation and recording by both evaluators (the principal investigator and another clinical nurse), which resulted in highly significant results in inter-rater reliability assessment.

The domains of “knowledge”, “execution” and “negotiation and use of health resources” obtained an rs value equal to 1, which reflects a perfect correlation. Therefore, there is total agreement between the two observers, possibly because the items that constitute this domain are more objective and more unanimously measured, obtaining equal results.

However, the same was not true for “self-monitoring”, “interpretation”, and “decision-making”, where we did not obtain perfect correlation, but we did obtain very acceptable and significant values. The fact that these domains present more subjective questions and require greater complexity in participant responses may be the basis for less objective measurement and explain the results obtained.

These results in inter-rater agreement assessment are in line with other studies that report that nursing interventions implemented in the interaction with patient/caregiver prioritize knowledge and capacity, being more focused on the health-disease transition^(15,16,21)^.

Including an item to assess preoperative (self-)care competency in the first part of the instrument allows the instrument to be used at any point during the transition process. The literature indicates that nursing interventions in the preoperative period ensure a better transition to the new life condition^(10)^. The development of instrumental learning in the preoperative period is fundamental for the development of (self)care competencies, and will be even more effective if it is structured^(10)^. Ostomy education initiated preoperatively improves recovery after surgery, promotes faster learning about ostomy care, reduces hospital stays, and reduces the severity and incidence of complications^(10)^.

Through the analysis of the literature consulted, although scarce, it was possible to detect a certain concern in selecting the best strategies to improve the quality of care for people with gastrostomies and their caregivers. It was concluded that there is still a long way to go toward systematization, improving the scope of interventions (pre-, post-operative, and follow-up), and supporting them with nursing-specific frameworks. The complexity of the changes that people and caregivers experience and the way each one faces and manages them must become the subject of explanation and analysis, using theoretical frameworks to substantiate them. There is significant work to be done in nursing care for people with gastrostomies and their caregivers^(22)^. Nurses must demonstrate a high level of interpersonal competencies, including the ability to involve a person and caregiver in decision-making, and is ideally positioned to empower (self)care and independence^(22)^.

Some authors advocate for standardized ways of documenting nursing care to help nurses support their interventions and, consequently, their assessment. They also argue that these plans should integrate a set of nursing interventions targeting bodily processes, with the aim of training for stoma (self-)care and preventing complications, as well as addressing adaptive processes^(21)^. The instrument now created is based on a set of nursing interventions structured towards training for (self)care, and can be used by nurses in their context of action.

The literature is inconsistent in its recommendations for nursing care for people with gastrostomies. Furthermore, it is not evidence-based, variable, and confusing. In searching for effective educational practices for (self)care for people with gastrostomies and their caregivers, we found some articles that address aspects such as indications, management, and complications^(23)^. Aspects such as perception, acceptance, and satisfaction remain controversial. The studies found reveal a greater concern with procedures and technical care^(22)^. Most studies do not address effective educational practices to develop self-care competencies in people with gastrostomies and their caregivers, thus revealing a gap in the literature. Living with a gastrostomy is known to affect habits and the need for adaptation to self-care; however, publications related to self-care competencies are scarce. Therefore, the newly created instrument emphasizes the need for nurses to better explore their role in promoting self-care and systematizing this care.

### Study limitations

This study aimed to describe the development process of a self-care competency monitoring instrument. However, and inherently, we also initiated its validity process, with content analysis by a group of experts and an interobserver reliability study. We know that assessing the psychometric properties of a developed instrument is strongly recommended^(11)^. However, this study is only part of a larger project that involves developing and validating an instrument to assess (self-)care for people with gastrostomy and their caregivers. Therefore, although this study does not include a complete analysis of the instrument validity and reliability, these psychometric properties will be assessed in future stages of this project. Furthermore, the pre-test was conducted to understand users’ (people with gastrostomy) and their caregivers’ perceptions of the instrument, avoiding terminology and pronunciation errors, revealing vague terms and potential misunderstandings, and thus integrating a small sample. However, it would be desirable for this sample to be more robust, with at least 30 participants. We also intend to apply the instrument to a more diverse sample from different areas of our country.

To reinforce the scientific rigor of the validity process of the newly created instrument, we will also act in accordance with the COnsensus-based Standards for the selection of health Measurement Instruments, a simplified guide on the measurement properties of assessment instruments based on international consensus^(24)^. This international guideline states that all measurement properties included in the taxonomy are relevant and should be assessed for any measuring instrument used in any application.

To study reliability, in addition to the inter-rater reliability already performed, we will use the assessment of internal consistency and the two-half test. The test-retest will be excluded, as it is related to the instrument temporal stability/reproducibility^(11)^. Since the main variable concerns (self-)care competency, it is not fixed in time. Therefore, it is expected to be continuously developed. The validity study consists of content, construct, and criterion validity. Content validity was ensured in the focus group with experts. Criterion validity cannot be assessed at this time, as there is currently no other valid instrument to assess the same or a similar concept^(12)^. It is expected that construct validity will be assessed through factor analysis and interdomain correlation, since they are related to form dimensionality confirmation and analysis of its theoretical structure^(11)^.

The instrument created is promising, but requires further validity to consolidate its use in clinical practice and research on this topic.

### Contributions to health, nursing or public policy

Early initiation of the transition process, supported by this new instrument, can enable continuous response to individuals’ and caregivers’ needs and can consolidate learning, promoting autonomy. The instrument can be used in any healthcare institution, at any stage of the development of (self-)care competency, allowing for the quantification and qualification of the different levels of this competency. The operationalization of indicators for the different domains in the instrument can contribute to the sharing of information between nurses and healthcare units, promoting continuity of care.

The instrument will thus prove very useful in the care process, constituting a tool that will enhance appropriate interventions to support a person and/or caregiver in developing their autonomy, preventing complications. Nurses, during nursing consultations with a person with a stoma or their caregiver, can use the instrument as a guide in training gastrostomy (self-)care competencies. The instrument can thus become a support tool in assessing the teaching/learning process by allowing for the systematization of information in the area of assessment and monitoring of (self-)care competencies as well as enabling continuity of care for patients/caregivers.

This instrument could also integrate with computer applications used to document nursing care in healthcare institutions, enabling rigorous and systematic recording of the development of (self-)care competencies for individuals with gastrostomies or their caregivers. By integrating information systems, it also enhances the creation of data for the development of new research on this topic. It could be a viable tool for managers and nurses to consider in the situational diagnosis of strengths and weaknesses in the development of (self-)care competencies. Thus, it can support nurses in decision-making and evidence-based management interventions, modifying care practices, facilitating the application of evidence in practice, and fostering an institutional culture that improves healthy transitions.

This instrument could provide consistency in care, as nurses are primarily responsible for direct care for people with gastrostomy and their caregivers, and has the potential to contribute to the continuous improvement of care provided to people with gastrostomy and their caregivers.

## CONCLUSIONS

Structured and continuous nursing interventions are visible in gains in quality of life and adaptation to the stoma, adding rigor and systematization to the person/caregiver dyad as a care partner for the health team and for the care contexts.

We believe that the newly created instrument “Self-care competency of people with gastrostomies and their caregivers” allows us to systematize all the information relevant to nurses’ clinical judgment regarding monitoring self-care competency and can serve as a fundamental tool in supporting the decision-making process. It allows us to quantify the different levels of self-care competency at any point in its development, from preoperative to late postoperative. This instrument can systematically produce indicators resulting from the nursing care provided to people with gastrostomies and their caregivers. Through it, we can identify more vulnerable individuals or caregivers at risk of developing an unadjusted transition process. This knowledge will be crucial for mobilizing available resources to meet the needs of people with gastrostomies and their caregivers.

However, future research is needed to validate this instrument to ensure greater safety, reliability, and validity for supporting nursing decision-making. We hope this instrument will stimulate further research in this field and lead to the emergence of new ways of researching content to support the adaptation process of people with gastrostomies, such as protocols or mobile applications.

## Data Availability

The research data are available within the article.
