# Supplementary material for: Instrument for assessing (self)care competence in people with gastrostomy and their caregivers: a methodological study
Source: Rev Bras Enferm. 2025 Dec 8;78(6):e20240484. doi: 10.1590/0034-7167-2024-0484 (PMC12707616; doi:10.1590/0034-7167-2024-0484)
Supplement: Translation

# Translation: Instrumento de avaliação da competência de (auto)cuidado na pessoa com gastrostomia e cuidador: estudo metodológico

**DOI:** 10.1590/0034-7167-2024-0484pt

**Authors:** Luzia Raquel Lopes Pinheiro, Maria Alice Correia Brito, Célia Samarina Vilaça Brito Santos

**Affiliations:** I Instituto Ciências Biomédicas Abel Salazar. Porto, Portugal; II Escola Superior de Enfermagem do Porto. Porto, Portugal

**Keywords:** Autocuidado, Estomaterapia, Gastrostomia, Jejunostomia, Enfermagem

## Abstract

**Objetivos:**

descrever o processo de construção de instrumento para monitorização do desenvolvimento da competência do (auto)cuidado na pessoa com gastrostomia e seu cuidador.

**Métodos:**

estudo de investigação metodológica. A validade de conteúdo foi obtida através de grupo de discussão de *experts*. O pré-teste do instrumento foi aplicado em amostra com 18 participantes, cuja técnica de amostragem foi não probabilística e intencional. Foi ainda avaliada a concordância entre avaliadores em hospital do norte de Portugal.

**Resultados:**

do estudo, resultou um instrumento com 34 indicadores de medida, agrupados em seis domínios, com reconhecida validade de conteúdo. No pré-teste do instrumento, foram obtidos bons valores de concordância entre avaliadores entre os 80-100%.

**Conclusões:**

o instrumento criado mostrou ser completo e de fácil aplicação na avaliação da competência de (auto)cuidado na ostomia de alimentação, sendo-lhe conferido potencial para futuras investigações da sua validade.

## INTRODUÇÃO

Considerada como uma das necessidades humanas básicas, a nutrição é essencial para a manutenção da vida, ocorrendo através dos processos de ingestão, digestão e absorção^(1)^. Entre os motivos associados aos desequilíbrios nutricionais, encontram-se lesões no sistema nervoso central, tais como acidente vascular cerebral, alterações da cavidade oral, da faringe e do esôfago (malformações, tumores, neoplasias, traumas químicos), paralisias (coma, acidente vascular cerebral, paralisia cerebral), traumatismos ou coordenação inadequada da mastigação e deglutição (cirurgias extensas do abdómen, lesões extensas do cérebro), após cirurgia radical da face e pescoço ou radioterapia/quimioterapia no trato digestivo superior^(2)^.

Apesar do grande avanço revelado pela nutrição parenteral, a via entérica ainda é a primeira escolha para suporte nutricional em pessoas com sistema digestivo funcionante, mas com incapacidade ou impossibilidade de alimentação por via oral. Estudos clínicos que comparam a eficácia da nutrição parentérica e da nutrição entérica concluem que a eficiência da via entérica é maior quando comparada com a parenteral^(1)^. As estomias de alimentação permitem manter o aporte nutricional a médio e longo prazo por via entérica^(2)^.

Existem diferentes tipos de estomias de alimentação, sendo o diagnóstico médico, a situação clínica/estado geral e o tempo de tratamento alguns dos aspectos que interferem na escolha do tipo de estomia que mais se adequa à pessoa^(1,2)^. São indicadas principalmente em situações em que a pessoa está impedida temporária ou definitivamente de se alimentar por via oral e possui a previsão de receber a nutrição entérica por período prolongado, normalmente superior a quatro semanas^(1-3)^.

Diante do aumento de tempo em que se evidencia a necessidade da descompressão digestiva ou do suporte alimentar, recomenda-se a realização da gastrostomia. Nas circunstâncias em que há impossibilidade de realização de uma gastrostomia, a jejunostomia é a alternativa escolhida^(4)^ (as gastrostomias, nomeadamente as endoscópicas percutâneas, são as mais frequentes, pelo que neste artigo usaremos o termo “gastrostomia”). A gastrostomia estabelece o acesso ao estômago através da parede abdominal. As técnicas habitualmente utilizadas para a realização da gastrostomia são a laparotomia (gastrostomia à Witzel, à Stamm e à Depage-Janeway) e a endoscopia (técnicas *Push, Pull* e Russell)^(4)^.

Independentemente do tipo de estomia, a pessoa fica sujeita a alterações profundas na sua imagem física e no seu estilo de vida, exigindo, assim, uma adaptação à sua nova condição, encarando novos desafios, sendo diversos os fatores que condicionam o processo de adaptação e facilitam ou dificultam a transição^(5)^. A competência para o (auto)cuidado se refere à habilidade complexa que os indivíduos aprendem para manter e promover a saúde física e mental, influenciando significativamente a adaptação do indivíduo e desempenhando um papel vital na reabilitação após a criação da estomia. Bons níveis de (auto)cuidado estão positivamente correlacionados com o estado geral de saúde e qualidade de vida^(6)^.

O desafio para a enfermagem está em ajudar a pessoa a realizar uma transição saudável para a sua nova condição de saúde, promovendo e reconstruindo sua autonomia diante do evento gerador de dependência, que é a existência de um estoma^(6,7)^. Também deve ser tida em conta a relevância da intervenção dos enfermeiros na preparação e no envolvimento do cuidador, assegurando o suporte adequado para o desenvolvimento de competências para prestação de cuidados à pessoa com estomia, promovendo, assim, uma transição saudável para o papel de cuidador^(7)^. Um número significativo de pessoas com gastrostomia é dependente para o (auto)cuidado, tendo de recorrer a um cuidador. As pessoas com estomias carecem de realizar uma série de procedimentos específicos para orientar regimes terapêuticos complexos, para manter o bem-estar e a qualidade de vida ao longo do tempo, para controlar os riscos, para gerir os sintomas da doença, e para reduzir a provável incidência de complicações^(6)^. Portanto, a promoção da competência de (auto)cuidado representa uma questão relevante nos cuidados de enfermagem às pessoas com estomias, tanto na investigação quanto na prática clínica. Permitirá, assim, compreender melhor os fatores que afetam o (auto)cuidado das pessoas com gastrostomia e implementar intervenções de promoção do (auto)cuidado eficazes. O acompanhamento metódico iniciado no período pré-operatório, continuado no pós-operatório e depois da alta hospitalar, promove a autoconfiança e a capacidade nos cuidados ao estoma, potencializando a adaptação psicossocial e melhorando a qualidade de vida^(6-8)^. Também as principais organizações internacionais de enfermeiros estomaterapeutas salientam esses aspectos, defendendo a necessidade de mais investigação para sustentar essas afirmações^(9,10)^.

É fundamental a criação de instrumentos que permitam aos enfermeiros, no campo de ação das suas intervenções autônomas, planejar e implementar cuidados através de ações estruturadas. Por um lado, instrumentos específicos e confiáveis que avaliem resultados são usados de forma a contribuir para transições saudáveis e melhor qualidade de vida das pessoas com estomia, mas, por outro, acrescentam conhecimento à disciplina e produzem indicadores de resultado. Até o momento, não se encontrou disponível nenhum instrumento para avaliar a competência do (auto)cuidado na pessoa com gastrostomia, sendo este aspecto propulsor deste estudo de investigação. Assim, o desenvolvimento de instrumento para avaliação do (auto)cuidado na pessoa com gastrostomia e cuidador, que possa ser utilizado pelos enfermeiros na avaliação de resultados sensíveis às suas intervenções e monitorizar as mudanças de comportamento nas pessoas ao longo de todo o processo de transição, será um contributo para a disciplina de enfermagem e indubitavelmente para a qualidade dos cuidados.

## OBJETIVOS

Descrever o processo de construção de instrumento para monitorização do desenvolvimento da competência do (auto)cuidado na pessoa com gastrostomia e seu cuidador.

## MÉTODOS

Neste subcapítulo, é realizada a descrição das etapas metodológicas do estudo.

### Aspectos éticos

O protocolo de pesquisa foi submetido à aprovação da comissão de ética de um hospital central do norte de Portugal com parecer favorável (documento n^o^ 237/2022). O processo de autorização seguiu os princípios éticos da pesquisa, incluindo as diretrizes da Declaração de Helsinque. Todas as informações recolhidas foram tratadas com exigente confidencialidade, sendo o acesso aos dados limitado aos investigadores autorizados para garantir a segurança e integridade dos mesmos. A fim de preservar a privacidade, nenhum dado identificável, como o nome, foi registrado, de forma a atestar a isenção de dano dos intervenientes, bem como a necessária proteção dos dados recolhidos.

### Desenho, período e local do estudo

Diante da natureza desta investigação e seu objetivo, desenvolveu-se estudo metodológico com uma abordagem qualitativa que engloba a construção de um novo instrumento de medida indispensável para produção de conhecimento na área em estudo^(11)^. Os dados foram recolhidos entre 1 fevereiro e 31 março de 2023 em hospital central do norte de Portugal. A presente pesquisa enquadra-se em estudo maior relativo à construção e validação de formulário para monitorização do “Desenvolvimento da competência de (auto)cuidado na pessoa com ostomia de alimentação e seu cuidador”.

### População, amostra, critérios de inclusão e exclusão

A população em estudo integra pessoas com gastrostomia e seus cuidadores que frequentam o serviço de gastroenterologia de hospital central do norte de Portugal. Da população referida, os critérios de inclusão no estudo foram ter idade igual ou superior a 18 anos, aceitar participar no estudo, ser portador de uma gastrostomia ou ser cuidador de uma pessoa com gastrostomia.

A técnica de amostragem selecionada foi a não probabilística intencional, o que permitiu aceder mais facilmente à população^(11)^.

### Protocolo do estudo

O estudo metodológico aqui apresentado compreende duas grandes etapas: a construção e a validação parcial do formulário criado. Para a construção e validação de conteúdo do instrumento de avaliação do desenvolvimento da competência do (auto)cuidado da pessoa com gastrostomia e do seu cuidador, utilizaram-se as etapas bem definidas pelos autores Lobiando-Wood e Haber^(12)^:

#### a) Definição do(s) conceito(s) que se pretendia(m) medir

Assim, iniciou-se a construção do instrumento com a definição dos seguintes conceitos centrais: autocuidado; competência; e gastrostomia. Através de revisão alargada de literatura usando os descritores “Autocuidado”, “Competência” e “Gastrostomia”, nas bases de dados MEDLINE, CINHAL, *Cochrane Clinical Answers*, MedicLatina, *Library, Information Science Technology Abstracts*, PubMed e livros de enfermagem de estomaterapia^(13,14)^, procuraram-se em cada um a definição e operacionalização do conceito de competência no (auto)cuidado à gastrostomia. Assim, definiu-se o conceito de competência no (auto)cuidado à gastrostomia, traduzindo-o para as dimensões e respectivos itens que o permitem mensurar. Procedeu-se à pesquisa de instrumentos de medida na área em estudo, não se encontrando até ao momento nenhum na área das gastrostomias. Os instrumentos encontrados se centram nas ostomias de eliminação.

#### b) Enumeração dos itens do instrumento que se poderiam constituir como indicadores passíveis de serem mensurados dos conceitos em análise

Para a disposição e seriação dos indicadores de resultado identificados no instrumento, foram utilizados como base de trabalho dois instrumentos pré-existentes:

- O formulário Competência do Autocuidado à Ostomia de Eliminação Intestinal (CAO-EI) da Escola Superior de Enfermagem do Porto (ESEP) (avaliação do desenvolvimento da competência no autocuidado à estomia de eliminação intestinal)^(15)^;
- O formulário Competência do Autocuidado à Ostomia de Ventilação (CAO-OV) da ESEP (avaliação do desenvolvimento de competência no autocuidado à ostomia respiratória)^(16)^.

Com a análise estatística dos resultados obtidos pela implementação do estudo piloto do formulário Competência do Autocuidado à Ostomia de Eliminação Intestinal (CAO-EI) na amostra de 180 participantes, o formulário obteve um alpha de Cronbach global de 0,93, apresentando, assim, boa consistência interna^(15)^.

A fidelidade do CAO-OV foi avaliada através do coeficiente alfa de Cronbach, demonstrando boa consistência interna (alfa=0,89) em amostra de 80 participantes e reconhecida validade de conteúdo^(16)^.

Diante da natureza multidimensional da competência no (auto)cuidado à gastrostomia, criamos um instrumento piloto com 33 indicadores, organizados em seis principais dimensões/domínios, como nos instrumentos pré-existentes^(15,16)^, nomeadamente: conhecimento; autovigilância; interpretação; tomada de decisão; execução; e negociação e utilização dos recursos de saúde.

No que se refere à escala de medida para avaliar a competência para o (auto)cuidado à gastrostomia, o instrumento proposto usa a atribuição de uma classificação ordinal, em escala Likert com cinco pontos, que permite avaliar os resultados alcançados de forma quantitativa e com melhor especificidade.

Na primeira parte do instrumento, acrescentou-se uma área para caracterização da pessoa com ostomia ou o seu cuidador, através das variáveis sociodemográficas (como a idade, condição civil, escolaridade e situação profissional), variáveis clínicas (diagnóstico clínico, tipo de cirurgia/procedimento) e variáveis de tratamento (consulta de enfermagem no pré-operatório, contato prévio com outras pessoas com gastrostomia).

#### c) Estudo da validade de conteúdo dos itens do instrumento

Para o estudo da validade de conteúdo do instrumento, optou-se por uma metodologia qualitativa, nomeadamente o grupo de discussão, que procurou incorporar os elementos do guia *COnsolidated criteria for REporting Qualitative research* como forma de conferir transparência, rigor metodológico, analítico e clareza contextual ao nosso estudo. A lista de verificação *COnsolidated criteria for REporting Qualitative research* consiste em 32 itens, organizados em três domínios: equipa de pesquisa; conceito do estudo; e análise/resultados^(17)^.

##### • Equipa de pesquisa

Os dados foram colhidos por uma investigadora em processo de doutoramento em ciências de enfermagem. A investigadora conduziu a reunião do grupo de discussão. A investigadora teve instrução das orientadoras, com experiência em investigação qualitativa, para a colheita de dados em estudos qualitativos, treinando a forma de colheita de dados neste tipo de estudo.

Foi estabelecido contato inicial com os participantes para apresentação da investigadora, seus objetivos e interesse pelo estudo. A investigadora não tinha relação prévia com os participantes.

Os participantes foram informados de que a investigadora estava conduzindo o estudo como parte de projeto maior de construção e validação de instrumento de monitorização da competência de (auto)cuidado.

##### • Conceito do estudo

A orientação metodológica declarada foi a análise de conteúdo através de grupo de discussão. O grupo de discussão é uma estratégia metodológica na qual existem a interação e a intervenção do investigador com um grupo de pessoas, *experts* na área em estudo, com o objetivo de procurar as suas interpretações profundas e os seus pontos de vista relativamente ao assunto em estudo. Consiste em discussão estruturada entre pessoas que dominam determinada matéria, centrada na interação grupal e no papel ativo do investigador como fomentador da discussão^(11)^. Com o propósito de garantir que os enfermeiros participantes possuíssem experiência na área em estudo, foram definidos os seguintes critérios de inclusão: trabalhar atualmente em consulta de enfermagem de estomaterapia ou serviço de gastroenterologia com pessoas com gastrostomia e cuidadores; ter pelo menos cinco anos de experiência nesses serviços; e ser docente/investigador na área do autocuidado e oncologia. Deveriam, ainda, aceitar participar no estudo de forma livre e consentida. A seleção dos participantes para o grupo de discussão ocorreu até o alcance de um grau de número aceitável de elementos. A amostra foi não probabilística e intencional, ocorrida após aceitação de participação no grupo de discussão. Foram convidados dez enfermeiros, e todos aceitaram participar no estudo. Não houve presença de ninguém além dos participantes e do investigador. Assim, o grupo de *experts* foi composto por dez profissionais, todos do sexo feminino, com média de idades de 45 anos, maioritariamente com especialidade na área médico-cirúrgica, e cinco com formação pós-graduada em estomaterapia, todos com mais de dez anos de experiência na área. O grupo integrou duas docentes de enfermagem (uma delas com investigação no âmbito do autocuidado e outra no âmbito da oncologia e estomaterapia) e sete enfermeiros da prática clínica (quatro enfermeiras estomaterapeutas a trabalhar na consulta de enfermagem de estomaterapia e três enfermeiros de serviços de gastroenterologia) que, em conjunto, avaliaram a coerência de cada indicador e a sua representatividade para o conceito a ser avaliado. A opção por este grupo de *experts* se prendeu ao fato de serem reconhecidos como detentores de maior nível de conhecimento disciplinar na área em estudo e trabalharem em hospitais da zona norte de Portugal. Foram convidados por contato telefônico, sendo informado o objetivo do estudo, e todos aceitaram participar (não se utilizou um documento formal de autorização, visto que a reunião foi realizada fora da instituição, em tempo livre dos profissionais).

##### • Análise e resultados

Assim, a versão inicial do instrumento e o respectivo manual de preenchimento (que explicitamos em seguida) foram previamente enviados por email a todos os elementos do grupo de *experts* para que os analisassem individualmente. Era recomendado que analisassem cada domínio, respectivos indicadores e critérios definidores da competência de (auto)cuidado, indicando se devia manter, alterar ou retirar e justificar a decisão. Posteriormente, foi agendada uma reunião, realizada *online* no dia 23 de outubro de 2023, pela dificuldade de disponibilidade presencial comum a todos. Nesta reunião, realizou-se conversação planificada e desenhada cuidadosamente, procurando obter informação pertinente sobre a área de interesse entre os avaliadores^(11)^. A reunião foi orientada pelo investigador. Como roteiro, utilizou-se o próprio instrumento, seguindo os indicadores de resultado e respectivos critérios definidores. Foi analisado cada indicador e respectivos critérios definidores, um por um, só avançando depois de concordância total (100%) entre *experts*. Os dados verbalizados durante a reunião pelos participantes foram transcritos para a ferramenta *Microsoft Word^®^*, não sendo usada gravação de áudio ou visual durante a reunião.

No final desta reunião, que durou cerca de duas horas, todos os *experts* intervenientes concordaram com os 33 indicadores dos diferentes domínios da competência e respectivos critérios definidores de cada um dos indicadores, depois de introduzidas algumas alterações na linguagem previamente utilizada. O instrumento final elaborado, já com todas as alterações e sugestões resultantes do grupo de discussão, foi compartilhado com todos os participantes por e-mail, solicitando resposta ao mesmo e recebendo *feedback* positivo dos dez participantes.

#### d) Criação do manual de preenchimento para os usuários do instrumento

Para garantir a correta e rigorosa aplicação do instrumento, foi criado um manual de preenchimento, em que são traçados de forma pormenorizada os critérios definidores de cada um dos indicadores dos seis domínios da competência de (auto)cuidado de forma teórica e conceptual. Portanto, é assegurado que os escores conferidos aos itens manifestem realmente as características definidoras dos indicadores da competência de autocuidado, independentemente de quem o aplique.

#### e) Pré-testagem e teste piloto dos itens/indicadores do instrumento

Quando uma primeira versão de um novo instrumento é formulada, é imprescindível garantir que este seja adequável e que responda efetivamente às perguntas colocadas pelo investigador^(11)^. Assim, um pré-teste de um novo instrumento possibilita perceber como as questões e respostas são percebidas, evitar equívocos de terminologia e de enunciação, permitindo ainda revelar termos vagos e possíveis incompreensões.

O instrumento foi, então, submetido a um pré-teste com um conjunto de 18 pessoas, nove pessoas com gastrostomia e nove cuidadores, em separado, acompanhados na consulta externa de acessos entéricos de gastroenterologia. Sobre o tamanho da amostra do pré-teste, concordam entre si autores^(18)^ que 15 a 30 respondentes são suficientes para o pré-teste. A presente pesquisa resulta de estudo maior de construção e validação de formulário para monitorização do “Desenvolvimento da competência de (auto)cuidado na pessoa com ostomia de alimentação e seu cuidador”, em que se prevê uma amostra final a rondar os 180 participantes. Assim, consideramos 18 pessoas para o pré-teste, que corresponde a 10% da amostra. O instrumento foi aplicado pelo investigador principal a cada um dos participantes do pré-teste, sendo-lhes pedido que respondessem aos itens formulados com respostas desenvolvidas/comentadas, descrevendo o significado que atribuíam às questões individualmente, bem como ao instrumento na sua globalidade, incluindo ainda as condições da sua aplicação.

O investigador principal realizou a observação de 15 consultas de enfermagem como método complementar ao pré-teste na instituição proposta, avaliando se os indicadores de cada domínio da competência de (auto)cuidado, definidos para o instrumento piloto, respondiam de forma integral ao pretendido. O investigador usou o próprio instrumento como roteiro para verificar se correspondiam na prática às intervenções de enfermagem implementadas no treino da competência de (auto)cuidado da pessoa com gastrostomia ou cuidador em contexto de consulta de enfermagem.

## RESULTADOS

Foi aceite como conceito multidimensional a competência de (auto)cuidado à gastrostomia e que este conceito integra seis domínios aos quais correspondem os respectivos indicadores de resultado. Foram organizados e sistematizados os indicadores pelos seis domínios da competência (conhecimento, autovigilância, interpretação, tomada de decisão, execução, e negociação e utilização dos recursos de saúde). A ordem dos domínios e dos respectivos indicadores revelou ser facilitador na aplicação do instrumento e mostraram ser relevantes para medir o construto em análise.

Assim, com o debate e análise do instrumento no grupo de discussão, emergiram as mudanças: utilização do termo “(auto)cuidado”; utilização do termo “pessoa com estoma”; utilização do termo “procedimento” e “cirurgia” e acrescentado o ponto “a aguardar cirurgia/procedimento” - tal como apresentadas no Quadro 1.

**Quadro 1:** Mudanças efetuadas no instrumento de acordo com o grupo de discussão

Versão inicial	Versão final
“Autocuidado”	(auto)cuidado: utilização do termo (auto)cuidado no título do instrumento visto este poder ser aplicado também ao cuidador.
“Doente portador de ostomia”	Pessoa com estoma.
“Teve contato com pessoas com um estomia antes da cirurgia?”	“Teve contato com pessoas com uma estomia antes da cirurgia/procedimento?”: considerando que as estomias de alimentação não são todas cirúrgicas, sendo realizadas maioritariamente por técnica endoscópica.
“Participou em consulta de enfermagem de estomaterapia no pré-operatório?”	“Participou em consulta de enfermagem no pré-operatório?”: (considerando que as pessoas com estomia de alimentação são em geral acompanhadas pela equipe de enfermagem dos serviços de gastroenterologia, sendo que apenas uma pequena parte é seguida na consulta de enfermagem de estomaterapia).
“Há quanto tempo foi realizada a cirurgia/procedimento para construção do estoma de alimentação?”	Incluiu-se na primeira parte do instrumento, no ponto sete “Há quanto tempo foi realizada a cirurgia/procedimento para construção do estoma de alimentação”, a alínea “A aguardar cirurgia/procedimento”, por o instrumento poder ser utilizado no pré-operatório.

Seguidamente, o instrumento e o seu respectivo manual foram devidamente corrigidos com as sugestões de linguagem sugeridas pelo grupo de discussão. Após esta análise e harmonização, foi concretizada a versão final do instrumento e do manual de apoio ao preenchimento do mesmo. Mantiveram-se as seis dimensões da competência de (auto)cuidado à gastrostomia, os indicadores e respectivos domínios da competência de (auto)cuidado à gastrostomia, que constavam no instrumento, que foram considerados pertinentes e adequados para medir o construto em análise.

Realizou-se a observação de 15 consultas de enfermagem para complementar os resultados obtidos no pré-teste. Durante a observação de 15 consultas de enfermagem, verificou-se que o enfermeiro abordava sempre a importância da higiene oral na pessoa com gastrostomia, e este item não constava no instrumento elaborado. Constatou-se, assim, a necessidade de incluir o item “Refere a importância da higiene oral”, acrescentando-o ao domínio do conhecimento da competência de (auto)cuidado à ostomia de alimentação.

O instrumento apresenta seis domínios da competência de auto(cuidado): conhecimento, autovigilância, interpretação, tomada de decisão, execução e negociação e utilização dos recursos de saúde - tal como apresentado no Quadro 2.

**Quadro 2:** Domínios da competência do (auto)cuidado do instrumento e respectivos indicadores

Domínios da competência	Indicadores
Conhecimento	Refere o que é um estoma de alimentação. Refere qual é a finalidade do estoma de alimentação. Refere as características do estoma de alimentação. Refere os sinais de complicação do estoma de alimentação. Refere quais os dispositivos necessários no cuidado ao estoma. Refere quando deve proceder à substituição da compressa/rede fixação. Reconhece a importância da higiene oral. Refere quais os recursos disponíveis na comunidade à pessoa ostomizada. Reconhece as suas necessidades na área do conhecimento sobre o cuidado ao estoma.
Autovigilância	Observa o estoma de alimentação. Identifica as características do estoma de alimentação. Identifica sinais de complicação do estoma de alimentação. Regista ocorrências significativas.
Interpretação	Questiona detalhadamente com o objetivo de encontrar uma explicação. Refere quais as possíveis causas de complicações do estoma de alimentação Reconhece que os resultados do autocuidado ao estoma influenciam o seu bem-estar.
Tomada de decisão	Estabelece prioridades na tomada de decisão. Reconhece as possíveis consequências das suas decisões. Previne as complicações da ostomia. Verbaliza o que fazer para minimizar as complicações da ostomia.
Execução	Executa os procedimentos, atendendo ao conforto. Executa os procedimentos para que o resultado seja esteticamente agradável e funcional. Gere o tempo na execução de procedimentos para obter os melhores resultados. Organiza o material necessário para o cuidado à ostomia. Lava a pele periestomal. Seca a pele periestomal. Coloca compressa tecido não tecido no estoma/rede fixação. Roda 360º a sonda botão de acordo com protocolo. Administra corretamente a alimentação. Administra corretamente a medicação. Lava a sonda no final de administrar a alimentação/medicação e verifica se a tampa se encontra fechada.
Negociação e utilização dos recursos de saúde	Negoceia os diferentes recursos disponíveis no apoio à pessoa ostomizada. Recorre aos serviços de saúde para esclarecimento de dúvidas e/ ou aconselhamento. Recorre oportunamente aos serviços de saúde/enfermeiro face a complicações da ostomia Avalia o cuidado prestado pelos serviços de saúde.

O manual de preenchimento foi elaborado de forma a clarificar e uniformizar o preenchimento do instrumento, manual este que contribuirá para o estudo da fidelidade inter-juízes. Assim, pretende-se, com este manual, que quaisquer enfermeiros instruídos e treinados chegarão aos mesmos resultados quando observam o nível da competência de (auto)cuidado.

Para se medir os indicadores mencionados nos diferentes domínios, usou-se uma escala de apreciação do tipo Likert, variando entre zero e cinco, em que quanto maior a pontuação, maior a competência no (auto)cuidado demonstrada. Assim, o zero corresponde a “não se aplica”; o um a “não demonstra”; o dois, três e quatro a “demonstra parcialmente” (de acordo com a percentagem de critérios definidos para o indicador) e o cinco a “demonstra totalmente” (Quadro 3).

**Quadro 3:** Pontuação da escala do tipo Likert e respetiva interpretação

Escala de Likert Pontuação	Interpretação
0	Não se aplica
1	Não demonstra
2	Demonstra parcialmente: cumprimento de cerca de 25% dos critérios apontados para o indicador do domínio da competência
3	Demonstra parcialmente: cumprimento de cerca de 50% dos critérios definidos para o indicador
4	Demonstra parcialmente: cumprimento de cerca de 75% dos critérios estabelecidos para o indicador do domínio da competência
5	Demonstra totalmente

Durante a realização do pré-teste, procedeu-se ainda a uma reflexão falada com as pessoas a quem se aplicou o instrumento, as quais não identificaram questões menos claras ou de difícil interpretação.

O instrumento desenvolvido foi um formulário, que apresenta estruturalmente semelhanças ao questionário, mas é preenchido pelo investigador. Assim, o formulário final mantém as seis dimensões da competência de autocuidado, passando a incluir um total de 34 itens. A avaliação desses 34 itens pressupõe que o investigador avalie questionando e observando a pessoa com gastrostomia e cuidador sobre o desenvolvimento da competência de (auto)cuidado, considerando os indicadores relativos a cada domínio da competência.

Para o estudo da concordância entre avaliadores ou também designada fidelidade inter-juízes, realizou-se uma comparação entre os dados obtidos pelo observador A *versus* os do observador B. Sobre o número de participantes para o estudo da concordância entre avaliadores^(11,18)^, definiu-se cerca de 10% da amostra. Os dois observadores aplicaram o formulário em simultâneo aos mesmos sujeitos, 14 cuidadores e sete pessoas com estoma de alimentação.

Através da análise do quadro, é possível constatar que existe uma correlação forte entre os resultados obtidos pelos diferentes avaliadores, sendo que os domínios de autovigilância, interpretação e tomada de decisão obtiveram um valor de rs acima dos 0,80, com um nível de significância de 0,001 (Quadro 4). Os domínios de “conhecimento”, “execução” e “negociação e utilização dos recursos de saúde” obtiveram o valor rs igual a 1, o que traduz uma correlação perfeita, existindo, portanto, concordância total entre os dois observadores (Quadro 4).

**Quadro 4:** Avaliação da concordância entre avaliadores utilizando o teste não-paramétrico de correlação de Spearman

	Conhecimento Avaliador B	Autovigilância Avaliador B	Interpretação Avaliador B	Tomada de decisão Avaliador B	Execução Avaliador B	Negociação Avaliador B
Conhecimento Avaliador A	1,000^**^					
Autovigilância Avaliador A		0,896^^**^^ ^p<0,001^				
Interpretação Avaliador A			0,802^^**^^ ^p<0,001^			
Tomada de decisão Avaliador A				0,847^**^ ^p<0,001^		
Execução Avaliador A					1,000^**^	
Negociação Avaliador A						1,000^**^

## DISCUSSÃO

A pessoa com gastrostomia deve estar preparada para o (auto)cuidado, visando ao seu empoderamento e autonomia. O empoderamento da pessoa com gastrostomia e do seu cuidador poderá interferir diretamente na redução de custos e na diminuição de complicações^(19)^. Assim o enfermeiro deve envolver a pessoa e/ou cuidador, sempre que possível, em todos os cuidados prestados^(19)^. É conhecido que o treino prático da competência de (auto)cuidado à gastrostomia, o apoio e o aconselhamento por enfermeiros ajudam a enfrentar efetivamente os desafios encontrados pelas pessoas e os seus cuidadores^(20)^.

A escassa produção científica sobre a avaliação especifica da competência de (auto)cuidado da pessoa com gastrostomia e do seu cuidador promoveu o investimento na elaboração deste instrumento. O enfermeiro poderá, assim, recolher dados rigorosos e completos, identificando as dificuldades sentidas pelas pessoas com gastrostomia e cuidadores no domínio da competência de (auto)cuidado. Este instrumento mostrou ser capaz de avaliar as necessidades no domínio da competência de (auto)cuidado, permitindo ao enfermeiro o diagnóstico de reais focos de atenção e intervenções ajustadas à melhoria contínua dos cuidados de enfermagem.

A primeira parte do instrumento que integra os dados sociodemográficos e as variáveis clínicas e de tratamento foi considerada pertinente para análise posterior da sua relação com a variável principal: competência de (auto)cuidado. Essas características devem ser conhecidas na medida em que poderão estar relacionadas ao progresso da competência de autocuidado e, no futuro, permitir a elaboração de hipóteses de investigação.

Assumimos o conceito da competência de (auto)cuidado como multidimensional, integrando seis dimensões (conhecimento, autovigilância, interpretação, tomada de decisão, execução, e negociação e utilização dos recursos de saúde)^(15,16)^. Esses domínios e respectivos indicadores traduzem o conceito que pretendemos medir.

O instrumento pode ser considerado longo, incluindo 34 indicadores. No entanto, tal é verificado pela necessidade de ser abrangente para contemplar um amplo conjunto de indicadores, suficiente para assegurar a correta monitorização do desenvolvimento dessa competência.

Após a reunião com o grupo de *experts*, foi possível realizar a reformulação do instrumento, garantindo, portanto, a congruência de cada indicador e a sua representatividade no conceito a ser avaliado.

Quanto à fidelidade inter-juízes, ao comparar os dados obtidos por dois avaliadores diferentes, verificou-se a existência de correlações perfeitas ou positivas altas, o que traduz elevada concordância. O manual de orientação de preenchimento do instrumento se revestiu de grande importância na observação direta e registro no instrumento pelos dois avaliadores (investigador principal e outro enfermeiro da prática clínica), que se traduziu na obtenção de resultados muito significativos na avaliação da fidelidade inter-juízes.

Os domínios de “conhecimento”, “execução” e “negociação e utilização dos recursos de saúde” obtiveram o valor rs igual a 1, o que traduz uma correlação perfeita, existindo, portanto, concordância total entre os dois observadores, possivelmente por os itens que constituem este domínio serem mais objetivos e de mensuração mais unânime, obtendo resultados iguais.

Porém, o mesmo não se verificou na “autovigilância”, “interpretação” e “tomada de decisão”, em que não obtivemos uma correlação perfeita, mas obtivemos valores muito aceitáveis e significativos. O fato de esses domínios apresentarem questões de cariz mais subjetivo e com uma maior complexidade na resposta do participante pode estar na base de uma mensuração menos objetiva e justificar os resultados obtidos.

Esses resultados na avaliação da concordância entre avaliadores vão de encontro a outros estudos que referem que as intervenções de enfermagem implementadas na interação com o doente/cuidador privilegiam o conhecimento e a capacidade, estando mais focalizadas na transição saúde-doença^(15,16,21)^.

Incluir, na primeira parte do instrumento, um item para avaliação da competência de (auto)cuidado no pré-operatório permite a utilização do instrumento em qualquer momento do processo de transição. A literatura indica que as intervenções de enfermagem no período pré-operatório garantem um melhor processo de transição para a nova condição de vida^(10)^. O desenvolvimento de aprendizagens instrumentais no pré-operatório é fundamental para evolução das competências do (auto)cuidado, e será ainda mais eficaz se for estruturada^(10)^. A educação sobre estomias iniciada no pré-operatório melhora a recuperação após a cirurgia, favorece a aprendizagem mais célere sobre os cuidados às estomias, reduz o tempo de internamento e diminui a gravidade e incidência das complicações^(10)^.

Pela análise da literatura consultada, ainda que escassa, foi possível perceber uma certa preocupação na seleção das melhores estratégias para acrescentar qualidade aos cuidados para as pessoas com gastrostomia e cuidadores. Foi possível concluir que ainda há um longo caminho a percorrer no sentido da sistematização, melhoria do alcance das intervenções (pré, pós-operatório e seguimento) e no seu suporte por referenciais próprios da enfermagem. A complexidade das mudanças que as pessoas e cuidadores vivenciam e o modo como cada um as enfrenta e gere devem se tornar objeto de explicação e de análise, recorrendo a referenciais teóricos que os substanciem. Há um trabalho significativo a ser constituído nos cuidados de enfermagem para as pessoas com gastrostomia e cuidadores^(22)^. O enfermeiro deve apresentar um alto nível de habilidades interpessoais, incluindo a capacidade de envolver a pessoa e cuidador na tomada de decisão, e está idealmente posicionado para capacitar para o (auto)cuidado e para a independência^(22)^.

Alguns autores defendem formas padronizadas de documentar os cuidados de enfermagem, de modo a ajudar os enfermeiros a sustentar a sua intervenção e, consequentemente, a sua avaliação. Também sustentam que esses planos devem integrar um conjunto de intervenções de enfermagem dirigidas aos processos corporais, no sentido da capacitação para o (auto)cuidado ao estoma e para a prevenção de complicações, assim como dirigidas aos processos adaptativos^(21)^. O instrumento agora criado assenta em um conjunto de intervenções de enfermagem estruturadas no sentido da capacitação para o (auto)cuidado, podendo ser usado pelos enfermeiros no seu contexto de ação.

A literatura é inconsistente em recomendações para os cuidados de enfermagem à pessoa com gastrostomia. Além disso, não é baseada em evidências, e é variável e confusa. Na procura sobre práticas educativas efetivas para o (auto)cuidado da pessoa com gastrostomia e seu cuidador, encontraram-se alguns artigos que abordam alguns aspectos como indicações, manuseamento e complicações^(23)^. Aspectos como percepção, aceitação e satisfação permanecem controversos. Os estudos encontrados revelam uma maior preocupação com procedimentos e cuidados técnicos^(22)^. Os trabalhos, na sua maioria, não abordam práticas educativas efetivas para desenvolver a competência do (auto)cuidado da pessoa com gastrostomia e seu cuidador, observando-se, desta forma, uma lacuna na literatura. Sabe-se que conviver com uma gastrostomia interfere nos hábitos e na necessidade de adaptação ao (auto)cuidado; porém, as publicações relacionadas à competência de autocuidado são escassas. Neste sentido, o instrumento agora criado pode enfatizar a necessidade de os enfermeiros explorarem melhor o seu papel na promoção do (auto)cuidado e na sistematização desses mesmos cuidados.

### Limitações do estudo

O objetivo deste estudo foi descrever o processo de construção de instrumento de monitorização da competência de autocuidado. No entanto, e por inerência, iniciamos também o seu processo de validação, com análise de conteúdo por um grupo de *experts* e estudo da fidelidade entre observadores. Sabemos que a avaliação das propriedades psicométricas de um instrumento construído é fortemente recomendada^(11)^. No entanto, este estudo é apenas uma parte de projeto maior que integra a construção e validação de instrumento para avaliação do (auto)cuidado a pessoas com gastrostomia e seus cuidadores. Assim, apesar de este estudo não incluir de forma desejável e completa a análise da validade e fidelidade do instrumento, essas propriedades psicométricas serão avaliadas em etapas futuras deste projeto. Por outro lado, o pré-teste foi realizado com a intenção de perceber a percepção dos utilizadores (pessoas com gastrostomia) e seus cuidadores sobre o instrumento, evitando erros de terminologia e enunciação, revelando termos vagos e possíveis mal-entendidos, integrando, portanto, uma amostra pequena. Seria, no entanto, desejável que essa amostra fosse mais robusta: com no mínimo 30 participantes. Também é nossa intenção a aplicação do instrumento a uma amostra mais diversificada e de diferentes áreas do nosso país.

Para reforçar o rigor científico do processo de validação do instrumento agora criado, atuar-se-á ainda de acordo com o *COnsensus-based Standards for the selection of health Measurement INstruments*, um guia simplificado sobre as propriedades de medida de instrumentos de avaliação baseado no consenso internacional^(24)^. Esta diretriz internacional refere que todas as propriedades de medição incluídas na taxonomia são relevantes e devem ser avaliadas para qualquer instrumento de medição usado em qualquer aplicação.

Para o estudo da confiabilidade, recorrer-se-à, além da fidelidade inter-juízes já realizada, à avaliação da consistência interna e do teste das duas metades. O teste-reteste será excluído, pois está relacionado à estabilidade temporal/reprodutibilidade do instrumento^(11)^. Como a variável principal diz respeito à competência no (auto)cuidado, esta não é estanque no tempo. Assim, é esperado que esta se encontre em contínuo desenvolvimento. O estudo da validade é constituído pela validade de conteúdo, de construto e de critério. A validade de conteúdo foi assegurada no grupo de discussão com *experts*. A validade de critério de momento não é possível avaliar, visto que ainda não existe outro instrumento válido para avaliar o mesmo conceito ou um conceito similar^(12)^. Prevê-se que a validade de construto seja avaliada através de análise fatorial e correlação interdomínios, uma vez que estão relacionados à confirmação da dimensionalidade do formulário e análise da sua estrutura teórica^(11)^.

O instrumento criado é promissor, mas exige validações complementares para consolidar o seu uso na prática clínica e na investigação sobre esta temática.

### Contribuições para as áreas da saúde, enfermagem ou políticas públicas

A iniciação antecipada do percurso de transição, com o suporte deste novo instrumento, poderá permitir responder às necessidades dos indivíduos e dos cuidadores de forma contínua e poderá consolidar as aprendizagens, promovendo autonomia. O instrumento poderá ser usado em qualquer instituição de saúde, em qualquer momento do desenvolvimento da competência de (auto)cuidado, permitindo quantificar e qualificar os diferentes níveis dessa competência. A operacionalização dos indicadores dos diferentes domínios no instrumento poderá contribuir para a partilha de informação entre enfermeiros ou unidades de saúde, promovendo a continuidade de cuidados.

O instrumento revelar-se-á assim muito útil no processo de cuidados, constituindo-se como uma ferramenta que potencializará intervenções adequadas para apoiar a pessoa e/ou cuidador a desenvolver a sua autonomia, prevenindo complicações. O enfermeiro, na consulta de enfermagem com a pessoa com estoma ou cuidador, pode utilizar o instrumento como guia no treino da competência de (auto)cuidado à gastrostomia. O instrumento pode se tornar, assim, uma ferramenta de apoio na avaliação do processo de ensino/aprendizagem, ao permitir sistematizar informação no domínio da avaliação e monitorização da competência de (auto)cuidado, assim como permitir a continuidade de cuidados ao doente/cuidador.

Este instrumento poderá, ainda, integrar os aplicativos informáticos em uso para documentação dos cuidados de enfermagem nas instituições de saúde, permitindo um registro rigoroso e sistemático do desenvolvimento da competência de (auto)cuidado da pessoa com gastrostomia ou cuidador. Ao integrar os sistemas de informação, potencia, também, a criação de dados para o desenvolvimento de novas pesquisas no âmbito dessa temática. Pode ser uma ferramenta viável a ser considerada pelos gestores e enfermeiros no diagnóstico situacional das potencialidades e fragilidades no desenvolvimento da competência de (auto)cuidado. Dessa forma, pode subsidiar os enfermeiros na tomada de decisão e nas intervenções da gestão baseadas em evidências, modificando práticas de cuidado, facilitando aplicação de evidências na prática e promovendo uma cultura institucional para melhorar as transições saudáveis.

Este instrumento poderá dar consistência aos cuidados, visto os enfermeiros serem os principais responsáveis pelos cuidados diretos à pessoa com gastrostomia e seus cuidadores, tendo potencial para ser o contributo para a melhoria contínua dos cuidados prestados às pessoas com gastrostomia e seus cuidadores.

## CONCLUSÕES

Intervenções de enfermagem estruturadas e contínuas são visíveis em ganhos na qualidade de vida e na adaptação à estomia, acrescentando rigor e sistematização sobre a díade pessoa/cuidador como parceira dos cuidados para a equipe de saúde e para os contextos de cuidados.

Cremos que o instrumento “Competência do (auto)cuidado da pessoa com gastrostomia e do seu cuidador” agora criado permite sistematizar toda a informação relevante ao juízo clínico do enfermeiro em termos de monitorização da competência de (auto)cuidado, podendo constituir-se como ferramenta fundamental no apoio ao processo de tomada de decisão. Permite quantificar os diferentes níveis da competência de (auto)cuidado em qualquer momento do seu processo de desenvolvimento, desde o pré-operatório até o pós-operatório tardio. Este instrumento poderá produzir, de forma sistemática, indicadores resultantes dos cuidados de enfermagem prestados às pessoas com gastrostomia e cuidadores. Através do mesmo, poder-se-á identificar pessoas ou cuidadores mais vulneráveis, em risco de desenvolverem um processo de transição não ajustado. Este conhecimento será basilar para mobilização de recursos disponíveis para satisfação das necessidades da pessoa com gastrostomia e cuidador.

Contudo, são necessárias investigações futuras que conduzam à validação deste instrumento, de forma a conferir maior segurança, fiabilidade e validade ao instrumento de apoio à tomada de decisão dos enfermeiros. Esperamos que este instrumento seja um estímulo para novas pesquisas neste domínio e que possam surgir novas formas de investigação de conteúdos de apoio ao processo de adaptação da pessoa com gastrostomia, como protocolos ou aplicativos móveis.

## Data Availability

Os dados da pesquisa estão disponíveis no corpo do artigo.
